# Supplementary material for: Empirical methods for controlling false positives and estimating confidence in ChIP-Seq peaks
Source: BMC Bioinformatics. 2008 Dec 5;9:523. doi: 10.1186/1471-2105-9-523 (PMC2628906; doi:10.1186/1471-2105-9-523)
Supplement: Additional File 1 — A variety of html documents from the USeq web site detailing the available applications, their best usage, output file type descriptions, command line menus, etc. [file 1471-2105-9-523-S1.zip › USeqUserGuides/JavaDoc.zip!/JavaDoc/index-files/index-14.html]

O-Index


|  |  |  |
| --- | --- | --- |
| **Overview** \| Package \| Class \| Use \| **Tree** \| **Deprecated** \| **Index** \| **Help** | |  |
| **PREV LETTER**   **NEXT LETTER** | **FRAMES**    **NO FRAMES**     **All Classes** |  |


A B C D E F G H I J L M N O P Q R S T U V W X Z 

---


## **O**

**objectArraytoAL(Object[])** - Static method in class gata.main.GATAUtil: Converts an Object[] to an ArrayList **objectArrayToArrayList(Object[])** - Static method in class util.gen.Misc: Converts an Object[] to an ArrayList **Oligo** - Class in trans.main: Container to hold information about a particular oligo. **Oligo(int, int, int, ArrayList, String)** - Constructor for class trans.main.Oligo: **OligoBase** - Class in bioroot.oligo: **OligoBase()** - Constructor for class bioroot.oligo.OligoBase: **OligoBean** - Class in bioroot.oligo: **OligoBean()** - Constructor for class bioroot.oligo.OligoBean: **OligoBean(int, DBUtil)** - Constructor for class bioroot.oligo.OligoBean: **OligoBean(ResultSet)** - Constructor for class bioroot.oligo.OligoBean: **OligoBean(String[], IdNameMatch[], IdNameMatch[], UserBean, DBUtil)** - Constructor for class bioroot.oligo.OligoBean: For bulk file upload should be 17? cells Name, Alias Oligo Sequence, Matching Gene Sequence, uM Stock Concentration, ng/uL Stock Concentration, Volume(uL), Purification, 0 1 2 3 4 5 6 7 Modifications, Orientation, Type, Storage Location, Availibility, VisibilityBean, Handling Instructions, Target Gene Name, Organism, Notes 8 9 10 11 12 13 14 15 16 17 **OligoFlipper** - Class in trans.tpmap: This reversed the oligo sequence and orientation in a tpmap file. **OligoFlipper(String[])** - Constructor for class trans.tpmap.OligoFlipper: **OligoIntensityPrinter** - Class in trans.main: Prints out the oligo intensity values, split by chromosome, as '.sgr' files for import into Affy's IGB. **OligoIntensityPrinter(String[])** - Constructor for class trans.main.OligoIntensityPrinter: **OligoMatch** - Class in bioroot.plasmid: **OligoMatch(BlastMatcher)** - Constructor for class bioroot.plasmid.OligoMatch: **OligoMatch(ResultSet)** - Constructor for class bioroot.plasmid.OligoMatch: **OligoModify** - Class in bioroot.oligo: Servlet to modify oligos using OligoBeans referenced from a Session object. **OligoModify()** - Constructor for class bioroot.oligo.OligoModify: **OligoSpreadSheet** - Class in bioroot.oligo: **OligoSpreadSheet()** - Constructor for class bioroot.oligo.OligoSpreadSheet: **OligoTiler** - Class in util.bio.seq: Tiles oligos across a list of genomic regions. **OligoTiler(String[])** - Constructor for class util.bio.seq.OligoTiler: **OligoUpload** - Class in bioroot.oligo: **OligoUpload()** - Constructor for class bioroot.oligo.OligoUpload: **OligoUploadBean** - Class in bioroot.oligo: Oligo bulk file upload bean. **OligoUploadBean()** - Constructor for class bioroot.oligo.OligoUploadBean: **OligoViewPreferences** - Class in bioroot.oligo: **OligoViewPreferences()** - Constructor for class bioroot.oligo.OligoViewPreferences: **optimizeAlignmentScore(String, String, int, int, int, int, int)** - Static method in class gata.main.GATAUtil: **optimizeScores(int[][])** - Method in class gata.aligner.LocalAlignment: **Option** - Class in util.apps.gump: **Option(String)** - Constructor for class util.apps.gump.Option: **orderAntibodies(String, UserBean, AntibodyBean[], DBUtil)** - Static method in class bioroot.antibody.AntibodySpreadSheet: Orders antibodys, sort of nasty, can't figure another way, reflection is way too slow. **ordered()** - Method in class trans.cel.ControlStats: **orderOligos(String, Preference, OligoBean[], DBUtil)** - Static method in class bioroot.oligo.OligoSpreadSheet: Orders oligos, sort of nasty, can't figure another way, reflection is way too slow. **orderPlasmids(String, UserBean, PlasmidBean[], DBUtil)** - Static method in class bioroot.plasmid.PlasmidSpreadSheet: Orders plasmids, sort of nasty, can't figure another way, reflection is way too slow. **orderStrains(String, UserBean, StrainBean[], DBUtil)** - Static method in class bioroot.strain.StrainSpreadSheet: Orders strains, sort of nasty, can't figure another way, reflection is way too slow. **Organism** - Class in bioroot: **Organism()** - Constructor for class bioroot.Organism: **OrganismBean** - Class in bioroot: **OrganismBean()** - Constructor for class bioroot.OrganismBean: **OrganismBean(int, DBUtil)** - Constructor for class bioroot.OrganismBean: **Organization** - Class in bioroot: Container for holding info about Organization membership. **Organization(DBUtil, int)** - Constructor for class bioroot.Organization: **overlap(ArrayList, int, int)** - Static method in class trans.anno.AnnotateRegions: Tests whether any startEnd int[] in the ArrayList of ints ovelaps a region defined by the startRegion and endRegion. **overlap(ArrayList, int, int)** - Static method in class trans.anno.AnnotateRegionsWithGeneList: Tests whether any startEnd int[] in the ArrayList of ints ovelaps a region defined by the startRegion and endRegion. **overlap(BindingRegion)** - Method in class trans.anno.BindingRegion: Returns -1 for no overlap, 0 for complete overlap, or a positive int for the # bases of overlap. **overlap(Interval)** - Method in class trans.anno.Region: Returns -1 for no overlap, or a positive int for the # bases of overlap. **overlap(GenomicRegion, int)** - Method in class trans.graphics.GenomicRegion: checks to see if regions overlap by the minimum maxGap, can set maxGap negative to require an overlap. **overlap(Interval, Interval)** - Method in class trans.main.OverlapCounter: checks to see if intervals overlap by the minimum maxGap, can set maxGap negative to require an overlap. **overlap(Window)** - Method in class trans.main.Window: **OverlapCounter** - Class in trans.main: Provides an intersection analysis on multiple Interval[]s. **OverlapCounter(String[])** - Constructor for class trans.main.OverlapCounter: **overlapOrAbut(SmoothingWindow, SmoothingWindow)** - Method in class edu.utah.seq.data.HeatMapMakerPosNeg: Assumes right window is to right of left or abuts or doesn't overlap. **overlapOrAbut(Window, Window)** - Method in class trans.main.WindowBlockMakerTwoColor: Assumes right window is to right of left or abuts or doesn't overlap. **overlapPeaks(Interval, Interval)** - Method in class trans.main.OverlapCounter: Returns distance between top peaks.

---


|  |  |  |
| --- | --- | --- |
| **Overview** \| Package \| Class \| Use \| **Tree** \| **Deprecated** \| **Index** \| **Help** | |  |
| **PREV LETTER**   **NEXT LETTER** | **FRAMES**    **NO FRAMES**     **All Classes** |  |


A B C D E F G H I J L M N O P Q R S T U V W X Z 

---
